# Authors’ Response to Peer Reviews of “Willingness to Pay for the COVID-19 Vaccine and Its Correlates in Bangladesh: Cross-Sectional Study”

**DOI:** 10.2196/79352

**Published:** 2025-08-15

**Authors:** Mohammad Bellal Hossain, Md Zakiul Alam, Md Syful Islam, Shafayat Sultan, Md Mahir Faysal, Sharmin Rima, Md Anwer Hossain, Abdullah Al Mamun, Abdullah- Al- Mamun

**Affiliations:** 1 Department of Population Sciences, University of Dhaka, Third Floor, Arts Faculty Building, Dhaka, 1000, Bangladesh, 880 01766517002; 2 Laboratory of Fertility and Wellbeing, Max Planck Institute for Demographic Research, Rostock, Germany; 3 Department of Social Relations, East West University, Dhaka, Bangladesh; 4 Department of Japanese Studies, University of Dhaka, Dhaka, Bangladesh

**Keywords:** Bangladesh, willingness to pay, vaccines, COVID-19, infectious diseases, infection control, public health, public safety, cross-sectional study, financial

*This is the authors’ response to peer-review reports for “Willingness to Pay for the COVID-19 Vaccine and Its Correlates in Bangladesh: Cross-Sectional Study.”*

## Round 1 Review

### Reviewer AT [1]

#### General Comments

*This paper [2] examines willingness to pay (WTP) for COVID-19 vaccines in Bangladesh using a cross-sectional survey. The integration of the health belief model and theory of planned behavior adds a theoretical foundation to the analysis. The study is well-structured, and the use of hierarchical logistic regression strengthens its analytical rigor.*

*However, several issues need to be addressed before acceptance. The sampling methodology raises concerns about representativeness, particularly due to the mix of online and face-to-face data collection.*

**Response:** Thank you for your insightful comment. We have detailed the process of sampling and data collection in the Methodology section (lines 114‐145). To minimize the bias of under- and overrepresentation, we have utilized the weight adjustment technique (lines 232‐241).

*Additionally, some statistical interpretations require further clarification, and the discussion on policy implications could be expanded to provide actionable recommendations. Addressing these concerns will enhance the overall impact and credibility of the study.*

**Response:** Thank you for your insightful comment. We have carefully interpreted the findings of our study (lines 259‐273) and provided actionable recommendations in the Conclusion section (lines 411‐429).

#### Specific Comments

##### Major Comments

*1. The study employs both online and face-to-face data collection. However, the online survey may have overrepresented educated and tech-savvy individuals, while the face-to-face survey followed quota sampling.*

**Response:** Thank you for your feedback. However, the digital divide in the country was considered when finalizing the methodology, and the ratio for face-to-face and online surveys was kept at 2:1, taking this into account. This is detailed in the Methodology section (lines 89‐90 and 98‐118).

*2. Clarify the adjusted odds ratio (aOR) interpretation. Some aOR values are close to 1, making practical significance questionable.*

**Response:** Thank you for your feedback. We have reanalyzed the data with weighted sample size to address your comment (Table 2).

*3. The impact of administrative divisions (eg, Sylhet having 4× higher WTP) should be further discussed. Are these differences due to economic, cultural, or policy variations?*

**Response:** Thank you for your valuable comment. In the discussion section, we further discuss the impact of administrative divisions (lines 355‐368).

*4. While the study suggests subsidized vaccination programs, it would be helpful to compare findings with other low- and middle-income countries’ WTP trends.*

**Response:** Several studies have been added to the discussion of low- and middle-income countries’ WTP trends (lines 318‐328).

##### Minor Comments

*5. Ensure table captions clearly describe what is presented (eg, Table 2 should explicitly state that it presents logistic regression results).*

**Response:** Thank you for your suggestion. We have made changes to the table captions to reflect what they present.

*6. Some sections contain grammatical errors and awkward phrasing (eg, “knowledge about the vaccine, vaccine process, conspiracy beliefs, behavioral practice, attitude toward a vaccine”; this list is repetitive and unclear).*

**Response:** Thank you for your insightful comment. We have revised the manuscript to minimize grammatical errors and improve awkward phrasing.

### Reviewer BN [3]

*This paper addresses an important and timely topic—WTP for COVID-19 vaccines in a developing country context. Understanding WTP is essential not only for informing current vaccine financing strategies but also for shaping policies related to equitable vaccine access in response to future public health challenges. The study is well-conceived and provides valuable insights into vaccine affordability and public perception in Bangladesh. With some refinements in presentation, statistical interpretation, and policy framing, the paper will be well-positioned for publication.*

*The abstract would benefit from being more concise and should more clearly highlight the key policy implications of the findings. Additionally, the statistical interpretation of the aORs requires careful attention. Several aORs are reported with values close to 1 (eg, family income aOR 1.0, P=.039; vaccine knowledge aOR 1.1, P=.003; behavioral practices aOR 1.1, P<.001), suggesting minimal effect sizes, yet they are statistically significant. While such significance may be driven by the large sample size, reporting CIs would allow for a more meaningful interpretation of the strength and direction of these associations.*

**Response:** Thank you for your valuable comment. We’ve rewritten the abstract to address your comment. We have also reanalyzed the data using a weighted sample size, which addresses the issue related to the aORs (Table 2) and reports CIs for more meaningful interpretation.

*The paper would also benefit from greater clarity around the construction of variables and the underlying measurement models. It is unclear how multiple survey items were combined to form factors such as knowledge, attitudes, and behavioral constructs. Using exploratory factor analysis could be beneficial to validate the grouping of items into coherent factors and strengthen construct validity. Providing factor loadings or at least a brief description of the item-grouping process would enhance the methodological transparency of the study.*

**Response:** Thank you for your insightful comment. However, please note that the objective of this paper is not to develop a scale on these issues. Instead, we aimed to identify the correlates of WTP. If we focus on the exploratory factor analysis and confirmatory factor analysis for these scales, the readers may get distracted. Thus, we focused only on the reliability analysis of the items used to measure these scales using Cronbach α, which has been discussed in the Methodology section (lines 123‐186).

*Another area for improvement involves the reporting of the income variable. In both Table 1 and Table 2, income appears to be modeled as a continuous variable, but the unit of measurement is not specified. Without this information, it is difficult to interpret an odds ratio of 1.0 meaningfully. If income is measured in small units (eg, Bangladeshi taka), the impact of each unit increase would be negligible. Categorizing income into meaningful brackets (eg, low, middle, high) and using those categories in logistic regression would make the results more interpretable and policy relevant.*

**Response:** Thank you for your comment. We’ve categorized the income variable, and the result now appears more interpretable and policy relevant (Tables 1 and 2).

*Additionally, the CIs for some variables in Table 2—such as income and COVID-19 vaccine conspiracy beliefs—appear to suggest nonsignificance, yet they are reported as significant. This inconsistency should be carefully reviewed and clarified.*

**Response:** Thank you for your valuable comment. We have categorized the income variable and revised the regression models to solve these issues (Table 2).

*Some of the measured constructs, such as knowledge and perceived susceptibility, show relatively low internal consistency (eg, Cronbach α of 0.612 and 0.657, respectively). It would be helpful for the authors to explain why these values are considered acceptable in this context or to discuss efforts made to improve reliability through item refinement or scale revision.*

**Response:** Thank you for your valuable comment. We’ve explained the issues of accepting low internal consistency in the Methodology section (lines 148‐153).

*Furthermore, the combination of nonprobability online sampling and quota sampling should be more clearly justified. While practical during a pandemic, it raises concerns about representativeness and potential sampling bias, which should be acknowledged more explicitly in the Discussion.*

**Response:** Thank you for your valuable feedback. We acknowledge the raised concern regarding the combination of nonprobability online sampling and face-to-face sampling. Our study employed this hybrid sampling method to ensure adherence to safety protocols amid the COVID-19 pandemic. Through an online survey, we quickly reached a large audience. However, later, we conducted face-to-face data collection using sampling criteria that ensured the representativeness of the sample, thereby determining the population’s national representation in terms of age, sex, residence, division, and marital status.

*The manuscript would also benefit from a thorough review for minor language and formatting issues. For instance, the phrase “explains explains” on page 13 should be corrected. Variable labels and descriptions in tables should be presented clearly and consistently.*

**Response:** Thank you for your comment. We have reviewed the manuscript and made the necessary corrections accordingly.

### Reviewer BM [4]

*1. In lines 79 and 80 of the manuscript [1], it is confusing why this wouldn’t be considered nationally representative if the data collection was conducted online.*

**Response:** Thanks for pointing this out. We were discussing the limitations of existing studies. We have now described the issues more carefully (lines 77‐81).

*2. As around 50% of the people are not interested in paying for the vaccine, this result should be considered with caution.*

**Response:** Thank you for your insightful comment. We acknowledge that WTP for a vaccine is context dependent. Our study’s results may be influenced by unique sociodemographic and cultural dynamics that appeared during data collection. We have mentioned this as a limitation in the Discussion section.

## Abbreviations

aOR: adjusted odds ratio
WTP: willingness to pay
